# Germline and somatic drivers in inherited hematologic malignancies

**DOI:** 10.3389/fonc.2023.1205855

**Published:** 2023-10-13

**Authors:** Julian Zoller, Despina Trajanova, Simone Feurstein

**Affiliations:** Department of Internal Medicine, Section of Hematology, Oncology & Rheumatology, University Hospital Heidelberg, Heidelberg, Germany

**Keywords:** inherited hematologic malignancies, next-generation sequencing somatic drivers, DDX41, ETV6, GATA2, RUNX1

## Abstract

Inherited hematologic malignancies are linked to a heterogenous group of genes, knowledge of which is rapidly expanding using panel-based next-generation sequencing (NGS) or whole-exome/whole-genome sequencing. Importantly, the penetrance for these syndromes is incomplete, and disease development, progression or transformation has critical clinical implications. With the earlier detection of healthy carriers and sequential monitoring of these patients, clonal hematopoiesis and somatic driver variants become significant factors in determining disease transformation/progression and timing of (preemptive) hematopoietic stem cell transplant in these patients. In this review, we shed light on the detection of probable germline predisposition alleles based on diagnostic/prognostic ‘somatic’ NGS panels. A multi-tier approach including variant allele frequency, bi-allelic inactivation, persistence of a variant upon clinical remission and mutational burden can indicate variants with high pre-test probability. We also discuss the shared underlying biology and frequency of germline and somatic variants affecting the same gene, specifically focusing on variants in *DDX41*, *ETV6*, *GATA2* and *RUNX1*. Germline variants in these genes are associated with a (specific) pattern or over-/underrepresentation of somatic molecular or cytogenetic alterations that may help identify the underlying germline syndrome and predict the course of disease in these individuals. This review is based on the current knowledge about somatic drivers in these four syndromes by integrating data from all published patients, thereby providing clinicians with valuable and concise information.

## Introduction

In 1999, Song et al. characterized the first inherited leukemia syndrome by recognizing that germline *RUNX1* variants lead to lifelong thrombocytopenia and an increased risk of myelodysplastic syndrome (MDS) and acute leukemia (AL) (1). Subsequently, many more genes have been associated with a germline predisposition to develop hematologic malignancies, which has led to the inclusion of the entity ‘myeloid neoplasms with germline predisposition’ into the revised 2016 World Health Classification (WHO) (2). In 2022, the WHO update incorporated additional newly associated genes and extended the phenotype of some of the syndromes to also include a predisposition to lymphoid malignancies (3). Generally, these syndromes are distinguished into three different categories (1): myeloid neoplasms without a preexisting disorder or organ dysfunction (e.g. *CEBPA*, *DDX41*), (2) myeloid neoplasms with a preexisting platelet disorder (*ANKRD26*, *ETV6*, *RUNX1*) and (3) myeloid neoplasms with potential other organ dysfunctions. The latter category covers a diverse spectrum of syndromes like bone marrow failure syndromes, telomere biology disorders and predisposition syndromes related to pathogenic variants in *GATA2*, *SAMD9* and *SAMD9L*. Likewise, the International Consensus Classification of myeloid neoplasms and acute leukemias in 2022 also encompassed hematologic neoplasms with germline predisposition in a similar format (4). Germline predisposition to hematologic malignancies has also been integrated in clinical guidelines such as the European LeukemiaNet and the National Comprehensive Cancer Network (5, 6). The most frequent germline syndromes are caused by pathogenic variants in transcription factors like *CEBPA*, *ETV6*, *GATA2*, and *RUNX1*, in the RNA helicase *DDX41*, and a variety of genes associated with telomere biology disorders and inherited bone marrow failure (7).

With the advent of diagnostic/prognostic next-generation sequencing (NGS) panels designed for somatic variants, germline variants are invariably detected as well, and recognition of these germline syndromes has increased. Since sequencing is performed on DNA from bone marrow/peripheral blood representing the affected tissue, the presence of a variant at a germline variant allele frequency (VAF) alone is not sufficient to presume germline origin. A multi-tier approach including confirmation in true germline material is usually required and specific criteria may indicate a higher likelihood for the presence of a germline variant.

Additional cytogenetic and molecular alterations are requisite for the transformation of a clone with a pathogenic germline variant to MDS or AL. These alterations may include (1): Additional well-known driver variants such as loss-of-function (LOF) variants in tumor suppressors, gain-of-function variants in (proto-) oncogenes and variants in genes involved in DNA repair, chromatin modification, transcriptional activation, DNA methylation and numerous others. Patients with congenital neutropenia represent an example for a strong association of somatic variants truncating the cytoplasmic domain of *CSF3R* with an impending leukemic transformation in variant carriers (8); (2) Contributory cytogenetic alterations that are well-known to promote disease. For instance, the association of germline *SAMD9/SAMD9L* variants with monosomy 7 is significant and essential for leukemogenesis in most patients (9, 10); (3) Bi-allelic inactivation with a second somatic hit on the other allele has been frequently observed in *DDX41, CEBPA, RUNX1*, *TP53*, and to a much lesser extent, in *GATA2* and is consistent with initiation or progression of disease (11–15). Additional factors such as an increase of the clonal VAF or an increase in somatic mutational burden also affect transformation or progression of disease (16–19).

Identifying the factors pointing to disease progression or transformation is critical for the follow-up of patients with germline syndromes and may affect the timing of treatment initiation, type of treatment and (preemptive) hematopoietic stem cell transplantation (HSCT). Current recommendations for patients with germline syndromes consist of a baseline bone marrow biopsy and additional bone marrow biopsies upon significant and persistent changes in blood counts (20). The timing and intervals of bone marrow biopsies and cytogenetic/molecular (re-) analyses may be tailored based on the individual risk of clonal alterations in these patients.

In this review, we discuss how germline variants can be identified using NGS-based panels primarily designed for somatic variants. We also assess somatic drivers in patients with germline variants in *DDX41*, *ETV6*, *GATA2* and *RUNX1* by reviewing the published evidence and analyzing the type and pattern of both germline and somatic variants in these genes and their role in malignant transformation.

## Germline variants detected upon diagnostic/prognostic sequencing of tumor tissue

The current standard of diagnosis and risk-stratification for myeloid and lymphoid malignancies are NGS panels based on a variety of genes known to be frequently mutated in the malignant clone. Risk-stratification elicited from molecular genetics are implemented in numerous risk scores, among others: the European LeukemiaNet risk classification for acute myeloid leukemia (AML) (5) and the Molecular International Prognostic Scoring System (IPSS-M) for MDS (16). These bear significant weight in estimating the patient’s individual risk and prognosis. Although we generally use the term ‘somatic NGS-panel’, these panels can also detect germline variants. Several studies have reported that NGS-based prognostic panels performed at the time of diagnosis frequently detect germline variants (14, 15, 21–32). The spectrum of germline mutated genes depends on the genes covered by the NGS panel and whether the panel has been designed to include copy number variants (CNVs), which also represent common predisposition alleles (33, 34). The following criteria indicate a higher likelihood of an underlying germline syndrome: (1) VAF — a heterozygous variant would be expected at/near heterozygosity (VAF 40-60%) and a recessive variant at/near homozygosity (VAF 90-100%) or in the compound heterozygous state. Of note, the VAF threshold may differ depending on the panel used so that the afore mentioned thresholds do not exclude the presence of a germline variant outside of this range (**Table 1**). (2) Presence of bi-allelic inactivation — specifically, tumor suppressors are often inactivated by a second hit on the *trans* allele, a phenomenon that has been well-described for *CEBPA*, *DDX41*, *RUNX1*, and *TP53* among others (**Table 1**) (11, 12, 14, 15, 37). (3) The persistence of a pathogenic/likely pathogenic variant at a germline VAF over the course of disease when remission is achieved is another clue and can be utilized when longitudinal sequencing data are present (**Table 1**). However, clonal hematopoiesis of unknown potential (CHIP) can also explain the persistence of a variant in remission/after therapy, when these variants were not part of the malignant clone. (4) Some variants are only reported in germline, for example LOF variants in *DDX41* are usually considered of germline origin (**Table 1**). The association of the mutational burden, defined as the total number of somatic variants in a patient, as independent prognostic variable with worsening outcomes has been well-established in MDS (16–18). The recently implicated IPSS-M concludes that the additional number of somatic variants in so called residual genes, that were not individually weighted, does lead to an additive increase in risk from 0 to 2, followed by saturation of worsening outcomes when 3 or more variants are present (16). A higher than usual mutational burden has been linked to a germline syndrome affecting the mismatch-specific DNA N-glycosylase MBD4. MBD4 acts as safeguard against damage from 5mC deamination and its deficiency results in increased risk to develop CHIP/AML, colorectal polyposis and uveal melanoma with significantly increased mutational burden compared to sporadic cases (35, 36). In AML specifically, MBD4-deficiency displays a 33-fold higher mutational burden with a unique mutational signature, where 95% of variants are CG>TG substitutions (**Table 1**) (35). Only few patients with autosomal recessive MBD4-deficiency and evidence of CHIP/AML have been described to date. In contrast to this rather distinct germline syndrome, most other germline syndromes are associated with a mutational burden similar to patients with sporadic disease affecting the same gene. Therefore higher, or lower mutational burden is not *per se* indicative of a germline syndrome.

**Table 1:** Germline variants identified in somatic diagnostic panels for hematologic malignancies.

Indication for an underlying germline variant	
(1) AD: VAF (het variant): 40-60%; AR: VAF (hom variant): 90-100% or comp het (2) Bi-allelic inactivation (3) Persistence of a pathogenic/likely pathogenic variant at germline VAF over the course of disease when remission is achieved (4) Variants only occurring in germline (e.g. LOF variants in *DDX41*) (21, 23, 25, 26, 32) (5) Significantly higher mutational burden, unique mutational signature (95% of variants are CG>TG substitutions) for variants in *MBD4* (35, 36)

AD, autosomal dominant; AR, autosomal recessive; comp het, compound heterozygous; het, heterozygous; hom, homozygous; LOF, loss-of-function; VAF, variant allele frequency.

Importantly, performing panel-based NGS designed for somatic variants cannot be used to rule out an underlying germline syndrome. Non-coding regions such as the 5’UTR in *ANKRD26*, containing all known germline predisposition alleles for this gene or the deep intronic enhancer in *GATA2* are not covered by a panel directed towards somatic variants. The same is true for CNVs, accounting for 10-15% of all predisposition alleles as mentioned earlier (33, 34), that are usually not detected either. Somatic and germline variant interpretation is based on a combination of different criteria (38, 39) and mechanisms of disease, location of variants and functional assessment may vary resulting in different evaluations of pathogenicity. **Table 1** summarizes the criteria suggesting a germline variant related to sequencing data and the genes with higher pre-test probability of variants confirmed in germline.

## Leukemogenesis in *DDX41* germline-mutated patients relies on bi-allelic inactivation of the *trans DDX41* allele

DDX41 is a multifunctional DEAD box helicase that operates as a DNA sensor, initiating an innate immune response, as a tumor suppressor through regulation of pre-mRNA splicing and RNA processing and as a modulator of gene expression of numerous oncogenes, tumor suppressor genes and genes involved in immune response and antigen presentation (40–42). *DDX41* germline variants cause the most frequent hematopoietic germline syndrome yet known (12, 43). Based on published studies of *DDX41* carriers, approximately 1.5% to 3.8% of unselected MDS/AML patients carry a germline *DDX41* variant (12, 43, 44), indicating that variants in this gene carry a high pre-test probability for a germline origin. Presumably somatic *DDX41* variants occur in approximately 2.4% of all MDS patients (16) and less than 1% of AML patients (45–47), strengthening the assumption that the majority of identified *DDX41* variants may indeed be germline.

**Figure 1:**
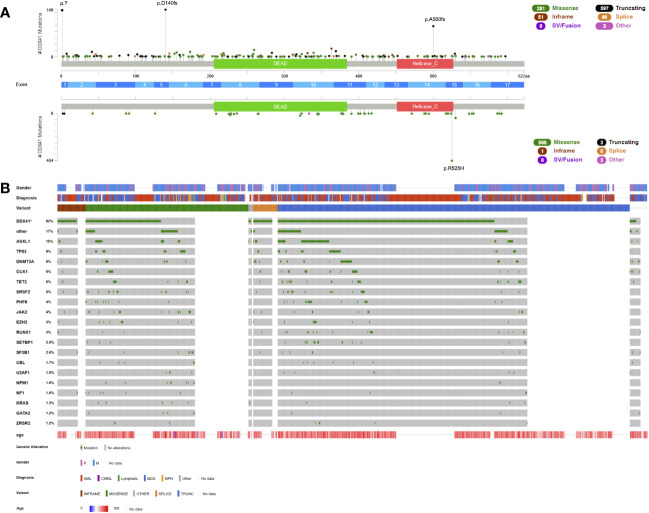
Lollipop plot and Oncoplot of all known *DDX41* germline variants. **(A)** Schematic of the *DDX41* transcript NM_016222.4 and its protein domains and exon distribution with the location of all reported variants. Germline variants are shown above the schematic and somatic variants below the schematic. The variant count is displayed on the x axis and the variant type is represented by different colors of the lollipops as outlined in the legend. **(B)** Oncoplot depicting *DDX41* variant carriers and their associated gender, diagnosis, age at diagnosis, variant type and most common co-occurring somatic variants including percentages. AML, acute myeloid leukemia; CMML, chronic myelomonocytic leukemia; MDS, myelodysplastic syndrome; MPN, myeloproliferative neoplasm. This schematic was created using cBioPortal (https://www.cbioportal.org/) (68, 69).

Based on a compilation of all published *DDX41* germline variants (12, 21, 22, 25, 33, 43, 44, 48–67), LOF variants, including nonsense, frameshift and canonical splice variants in *DDX41* amass to the majority of germline LOF variants (65.4%, n=641, **Figure 1A**). Almost all LOF variants (98,1%, n=629) occur early in the gene and are predicted or confirmed to undergo nonsense-mediated decay (NMD) (**Figure 1A**) with no functional protein product. Based on population data (https://gnomad.broadinstitute.org/) two founder variants, p.? (also known as p.M1I or p.M1?, NM_016222.4) and p.D140fs are common in the European (Non-Finnish) population at a VAF of 0.000156 and 0.000185, respectively, and p.A500fs is a founder variant most often detected in the East Asian subpopulation with a slightly lower VAF of 0.000109. Together, these three founder variants account for 39.9% (n=391) of all described germline patients so far (**Figure 1A**). Other variants like nonsynonymous substitutions (n=280, 28.6%) and indels (n=51, 5.2%) are also well-known predisposition alleles (**Figure 1A**). Few patients have been reported with non-canonical splice variants and one CNV has been described, encompassing exons 12 to 17 (67).

Germline *DDX41* variants predispose to myeloid malignancies, most often MDS and AML and to a lesser extent chronic myelomonocytic leukemia (CMML) or myeloproliferative neoplasms (MPNs) (**Figure 1B**). Lymphoid malignancies such as chronic lymphatic leukemia (65), large granular lymphocytic leukemia (62), lymphoplasmacytic lymphoma (65), plasma cell disorders (62, 65) and others have been reported in few patients with *DDX41* germline variants (**Figure 1B**). However, a causative association has not been clearly established. The median age of onset — unlike most other germline syndromes — is similar to the expected disease onset in sporadic disease: 67 years (n=328) in *DDX41* germline mutated AML patients and 68 years (n=326) in MDS patients (**Figure 1B**), which is congruent with the average age of onset for AML (68 years, https://seer.cancer.gov/statfacts/html/amyl.html) and MDS (71 years) patients (70). It is rarely diagnosed in patients before the age of 40 (1.6%, **Figure 1B**). The penetrance is reduced and currently estimated at roughly 50% (12). This germline syndrome often goes unnoticed as a result of this reduced penetrance, older age at diagnosis, and consequently fewer families with a positive family history.

Hematopoietic malignancies with a *DDX41* variant — irrespective whether this is a germline or somatic variant — define a distinct subgroup with a lower allelic burden, frequent association with a normal karyotype (**Table 2**) and overall more favorable prognosis (12, 44, 64, 65, 71). The favorable prognosis is retained in patients with otherwise poor prognostic markers such as multi-hit *TP53* variants when compared to age-matched controls (12, 43, 64). The disease shows a clear male predominance with a penetrance of hematologic malignancies that appears three-times higher in males than females (**Figure 1B**) (12, 25, 43).

**Table 2:** Somatic drivers in patients with germline *DDX41*, *ETV6*, *GATA2* and *RUNX1* variants.

	*DDX41*	*ETV6*	*GATA2*	*RUNX1*
Bi-allelic inactivation	Very common, 82% patients acquire a somatic hit in *DDX41*	Rare	6% acquire a somatic hit in *GATA2*	Common, 26% patients acquire a somatic hit in *RUNX1*
Molecular variants	Lower mutational burden, most common co-occurring somatic variants: *ASXL1* (15%), *TP53* (9%), *DNMT3A* (8%), *CUX1* (6%), *TET2* (6%) and *SRSF2* (5%)	Unspecific	Most common co-occurring somatic variants: *ASXL1* (39%), *STAG2* (34%), *SETBP1* (16%), *BCOR* (7%), *RUNX1* (7%), and *EZH2* (6%)	Most common co-occurring somatic variants: *TET2* (15%), *FLT3* as ITD or TKD variant (12%), *SRSF2* (12%), *ASXL1* (11%), *WT1* (8%), *DNMT3A* (8%), *BCOR* (8%) and *BCORL1* (8%)
Cytogenetic alterations	Most often normal karyotype (76.3%)	Normal or hyperdiploid karyotype, consistent with B-ALL as most common phenotype	Most common cytogenetic alterations: monosomy 7/del(7q) (37.8%), trisomy 8 (24.5%) and der(1;7)(q10;p10) (16%)	Unspecific

B-ALL, B-cell acute lymphatic leukemia; ITD, internal tandem duplication; TKD, tyrosine kinase domain.

Heterozygous *DDX41* LOF, however, is not sufficient to initiate leukemogenesis, and the majority of AML and MDS patients with germline variants carry additional somatic nonsynonymous substitutions primarily in the helicase catalytic center of the other allele. Approximately 82% of all patients with germline *DDX41* variants acquire a somatic hit with a mean VAF of 11%, 68.8% of those variants are located within the helicase domain and 19.7% within the DEAD box domain (**Figure 1A**, **Table 2**). The most common variant is p.R525H found in 71.7% of patients with a somatic hit and located within the helicase domain (**Figure 1A**). This hotspot variant perturbs ATPase activity and interferes with cell growth in a dominant-negative manner (72). Other common somatic variants include nonsynonymous substitutions p.T227M, p.P321L, p.E345D and p.G530D/S (**Figure 1A**). Curiously, while almost all LOF variants were confirmed germline with a somatic nonsynonymous substitution, three cases of an identical germline substitution within the DEAD box, p.R369G, with a confirmed LOF somatic *DDX41* variant (p.E2* and p.S4*) have been described (12, 25, 44).

The most common co-occurring somatic variants were found in *ASXL1* (15%), *TP53* (9%), *DNMT3A* (8%), *CUX1* (6%), *TET2* (6%) and *SRSF2* (5%) (**Figure 1B**, **Table 2**). Recurrent molecular markers such as variants in *NMP1*, *CEBPA* or internal tandem duplications (ITDs) of *FLT3* have only been described in few patients (12, 65). A normal karyotype was described in most patients for whom cytogenetic data were available (76.3%, n=242, **Table 2**). A range of different cytogenetic alterations e.g. del(5q), -7/del(7q), +8, del (20q), loss of chromosome Y and others, all frequently reported in MDS and AML with myelodysplasia-related changes, has been detected in the remaining patients. A complex karyotype was reported in several patients, most often including a del(5q), presumably derived from clonal evolution of a pre-existing del(5q) clone (12, 44, 65). Recurrent, subgroup-defining cytogenetic aberrations have only been described in four cases, two patients with inversion inv(16) (43, 65) and another two with a translocation t(8;21) (43, 65), again highlighting the near-absence of recurrent molecular or cytogenetic aberrations in *DDX41* germline-mutated patients. Little is known about clonal evolution in healthy carriers since most cases reported in the literature are already diseased. With the high percentage of bi-allelic inactivation of *DDX41*, low mutational burden and frequent absence of cytogenetic alterations, it stands to argue that bi-allelic inactivation is the main driver of disease. Prodromal features such as cytopenia with or without clonal markers and/or some level of dysplasia not meeting MDS criteria (yet) are infrequent (**Figure 1B**) and usually lead to an MDS/AML diagnosis down the line.

## ETV6-deficiency is associated with predominantly lymphoid malignancies concomitant with a normal or hyperdiploid karyotype

ETV6, located on chromosome 12p13, is part of the large ETS transcription factor family, comprising 28 genes primarily controlling tumor initiation and development. Its functional domains consist of a highly conserved N-terminal PNT domain involved in protein-protein interactions with itself and other proteins including FLI1, another member of the ETS family implicated in megakaryocyte lineage commitment (73). A central regulatory domain mediates repressive complex recruitment (including SMRT, Sin3A and NCOR) and autoinhibitory activity (74), and the C-terminal ETS domain conveys DNA binding (75). In cancer, structural variants of this gene are common and occur in a wide variety of different hematologic and solid tumors — with more than 30 translocation partners known to date (76). The well-known recurrent translocation t(12, 21)(p13;q22), resulting in a *RUNX1-ETV6* fusion, is identified in 20-25% of pediatric acute lymphatic leukemia (ALL) (77). Somatic variants of this gene are less frequent than structural alterations and have been reported in up to 5% of patients with T-ALL (78, 79), 2.7% of patients with MDS (16) and 1.1% in AML (39, 45, 46). Heterozygous germline variants of this gene were first reported in 2015 and go along with lifelong thrombocytopenia and a predisposition to both lymphoid and myeloid malignancies (80). The manner of inheritance is autosomal dominant with a near-complete penetrance exceeding 90% for thrombocytopenia but incomplete penetrance for hematologic malignancies, estimated in the range of 30% (75, 81). Based on the published studies to date (58, 75, 82–93), B-ALL is the most common malignant phenotype (n=26, 20%), followed by MDS/AML (n=8, 6.2%). At least two cases of mixed phenotype leukemia have been described (80, 82). There is no clear causal association between the few cases of patients with germline *ETV6* variants diagnosed with diffuse large B-cell lymphoma and polycythemia vera (83, 85, 94). The median age at onset of a hematologic malignancy is 11 years. Thrombocytopenia is present in most individuals from birth — albeit at times only recognized later in life — and when present is of moderate severity (median of 85+/-28 G/l) accompanied with a mild to moderate bleeding propensity. Nonsynonymous substitutions and LOF variants, predicted to undergo NMD, depict most of the variants reported to date (n=85, 65.9% and n=31, 24%, respectively, **Figure S1**). The nonsynonymous substitution, p.P214L (NM_001987.5), represents the most common recurrent variant, located within the central region, while other substitutions are mostly scattered across the C-terminal ETS domain (**Figure S1**). Intragenic CNVs have been described in two families, spanning exons 2 and 5 (84, 89). A constitutional balanced translocation t (12,14)(p13.2;q23.1) was identified in one family with familial B-ALL without thrombocytopenia and breakpoints were located in intron 1 of *ETV6* and *RTN1*, respectively (86). Bi-allelic inactivation of *ETV6* in the leukemic clone is rare, be it somatic variants or cytogenetic deletions of the *trans* allele and have only been described in few cases (**Table 2**) (82, 95). Karyotypic abnormalities often include a normal or hyperdiploid karyotype, consistent with common alterations detected in (childhood) B-ALL (**Table 2**). The pattern of somatic co-occurring variants in the leukemic clone resembles a typical pattern seen in ALL or AML/MDS without any obvious association of specific molecular or cytogenetic alterations with germline ETV6 deficiency (**Table 2**). A longitudinal study of four *ETV6* germline mutated carriers did not reveal any evidence of clonal hematopoiesis however, the number of patients was too small to draw conclusions at this time (88).

## Somatic *ASXL1*-, *STAG2*- and *SETBP1* variants, monosomy 7/del(7q) and trisomy 8 drive myeloid malignancies in GATA2-deficiency

GATA2 is a key zinc-finger transcription factor encompassing two zinc finger domains that regulate hematopoietic stem and progenitor cell self-renewal, survival, and differentiation (96). Somatic *GATA2* variants occur in 1.9% of all MDS patients (16) and 3.3% of all AML patients (39, 45, 46), mainly affect the first zinc finger domain and often co-occur with *CEBPA* variants. In contrast, *GATA2* germline variants are frequently found within the second zinc finger domain. These variants are common in childhood MDS, accounting for 15% of advanced and 7% of all primary pediatric MDS cases (97). GATA2-deficiency is strongly associated with monosomy 7 and is observed in more than half of all childhood MDS cases with monosomy 7 (97). Roughly 4 to 5% of MDS patients diagnosed as young adults carry *GATA2* germline variants (34, 98), however, the likelihood of a germline GATA2-deficiency decreases significantly with the age at diagnosis. More than half of all variants arise *de novo*, resulting in the lack of a positive family history and evidence of segregation among affected family members (11, 99). The phenotype is highly variable and includes hematopoietic and non-hematopoietic features, encompassing immunodeficiency predominantly through reduced or absent monocytes, B cells, natural killer cells, neutrophils, and/or dendritic cells, MDS/AML, pulmonary disease, vascular/lymphatic dysfunction, and hearing loss (11, 100). The penetrance for any phenotypic features is near-complete, whereas penetrance for myeloid malignancies is variable and incomplete, and most likely lies within a range of 30 to 75% (10, 97, 101, 102).

**Figure 2:**
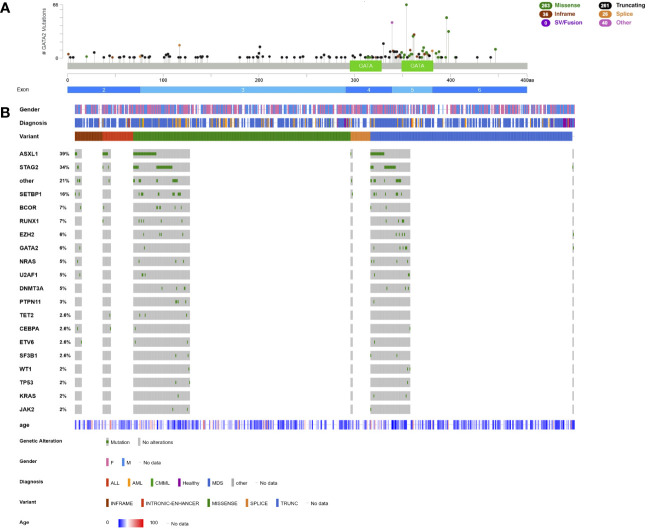
Lollipop plot and Oncoplot of all known *GATA2* germline variants. **(A)** Schematic of the *GATA2* transcript NM_032638.5 and its protein domains and exon distribution with the location of all reported germline variants. The variant count is displayed on the x axis and the variant type is represented by different colors of the lollipops as outlined in the legend. **(B)** Oncoplot depicting *GATA2* variant carriers and their associated gender, diagnosis, age at diagnosis, variant type and most common co-occurring somatic variants including percentages. ALL, acute lymphatic leukemia; AML, acute myeloid leukemia; CMML, chronic myelomonocytic leukemia; MDS, myelodysplastic syndrome. This schematic was created using cBioPortal (https://www.cbioportal.org/) (68, 69).

Based on published *GATA2* germline variants (11, 58, 89, 103–112), most variants are truncating LOF variants (46%, n=296) or nonsynonymous variants (44.1%, n=284, **Figure 2A**). CNVs account for 3.2% (n=20), with most of them being whole-gene deletions (21, 97, 104, 113–119). As opposed to somatic nonsynonymous variants located mainly within the first zinc finger domain, germline nonsynonymous substitutions are — with few exceptions — located within the second zinc finger domain (**Figure 2A**). Variants in the first versus second zinc fingers seem to harbour different functional consequences and co-occur with specific leukemic lesions (96). Hotspot amino acids within the second zinc finger are C349, C352, T354, T357, T358, L359, W360, R361, N371, A372, C373, L375, P385, M388, R396, and R398 (**Figure 2A**). A unique mechanism of disease are variants within a deep-intronic +9.5 44bp intronic enhancer element, consisting of an e-box, GATA and Ets/FLI1 motif and two spacers. Currently, causative variants have only been described in the e-box and ets/FLI1 motifs and make up roughly 6% of *GATA2* germline variants (97, 120), abrogating normal steady-state hematopoiesis and embryonic development (121).

Most patients with GATA2-deficiency have been diagnosed with MDS, CMML/juvenile myelomonocytic leukemia, MDS/MPN overlap, AML and bone marrow failure (78.3%, n=508, **Figure 2B**). The median age of onset for *GATA2*-related myeloid disease is 17 years (n=426, **Figure 2B**). Few cases of B- or T-ALL (105, 116, 122–124) and primary myelofibrosis (112) have been described (**Figure 2B**).

The most common co-occurring somatic variants are found in *ASXL1* (39%), *STAG2* (34%), *SETBP1* (16%), *BCOR* (7%), *RUNX1* (7%), and *EZH2* (6%, **Figure 2B**, **Table 2**). A second hit on the other *GATA2* allele was reported in 6% of patients (**Figure 2B**, **Table 2**). Compared to sporadic AML/MDS cases, variants in *ASXL1*, *SETBP1* and *STAG2* are statistically overrepresented in GATA2-deficiency, while variants in DNA methylation modifiers *DNMT3A* and *TET2* seem to be less common (**Figure 2B**). Monosomy 7/del(7q) is the most common cytogenetic alteration and is found in 37.8% of patients (n=165), followed by trisomy 8 in 24.5% (n=107) of cases (**Table 2**). A derivative chromosome der(1;7)(q10;p10), resulting in an unbalanced chromosomal translocation with trisomy 1q and deletion 7q, is also commonly observed in GATA2-deficiency (16%, n=35, **Table 2**). In contrast to monosomy 7/del(7q) and trisomy 8, the der(1;7) is a rare chromosomal alteration and significantly enriched in patients with GATA2-deficiency (109). *ASXL1*- and *STAG2* variants as well as monosomy 7 and trisomy 8 have been observed in several patients with no overt hematologic malignancy, however, these are also common driver variants in the patients with hematologic malignancies.

## Germline *RUNX1* variants frequently display bi-allelic inactivation in the malignant clone

The master transcription factor RUNX1 is located on chromosome 21q22 and acts as transcriptional regulator of normal hematopoiesis. Functional domains comprise a highly conserved Runt-homology domain (RHD) spanning 128 amino acids, a C-terminal transactivation and inhibitory domain and a VWRPY motif binding transcriptional repressors (125). Three main isoforms A, B, and C are expressed by the use of two promotors and alternative splicing and display isoform-specific functions controlling stem cell expansion and hematopoietic differentiation (126–128).

*RUNX1* is frequently mutated in myeloid malignancies in approximately 14.2% of patients with MDS (16) and 10.5% of patients with AML (45–47). *RUNX1* variants are considered poor prognostic factors in both MDS and AML and HSCT is mandated whenever possible (5, 16). Structural variants involving *RUNX1* are frequent, in fact *RUNX1* is the most common target of chromosomal translocations found in AL. The translocation t(8;21)(q22;q22) resulting in a *RUNX1-RUNX1T1* fusion is a recurrent genetic abnormality in AML and accompanied by a favorable prognosis (5). The above mentioned translocation t (12; 21), giving rise to a *ETV6*-*RUNX1* fusion protein defines a subtype of pediatric ALL and various other *RUNX1* translocation partners have been described as well (125).

Germline *RUNX1* variants were first linked to inherited thrombocytopenia and predisposition to myeloid malignancies in 1999 by Song et al. (1). It is currently estimated that in approximately 16% of *RUNX1*-mutated AML patients the identified *RUNX1* variant is indeed of germline origin — higher percentages were reported but germline pathogenicity was not established for all variants (15, 29). This leads to the occurrence of a germline *RUNX1* variant in about 1 to 2% of an unselected AML population (29, 31). The penetrance is near-complete for mild to moderate thrombocytopenia with normal sized platelets, potentially in combination with an additional bleeding propensity caused by platelet alpha or dense granule secretion defects and/or impaired platelet aggregation (129). Several patients with intermittent or transient thrombocytopenia have been described so that serial assessment of the platelet count should be considered in patients with presumed normal platelet count. The penetrance for hematologic malignancies is incomplete and estimated at about 40-50% (130, 131).

**Figure 3:**
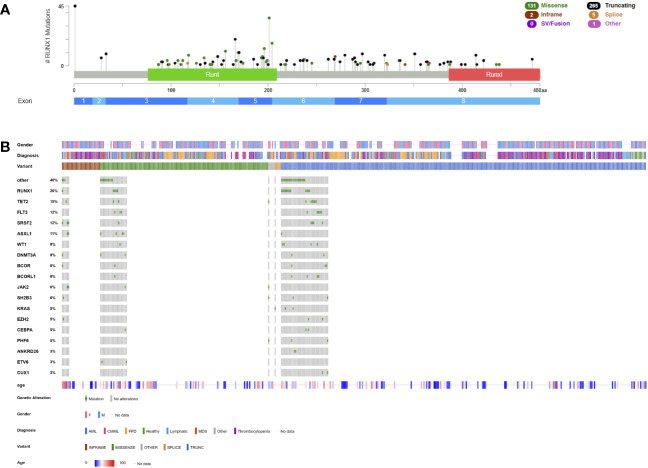
Lollipop plot and Oncoplot of all known *RUNX1* germline variants. **(A)** Schematic of the *RUNX1* transcript NM_032638.5 and its protein domains and exon distribution with the location of all reported germline variants. The variant count is displayed on the x axis and the variant type is represented by different colors of the lollipops as outlined in the legend. **(B)** Oncoplot depicting *RUNX1* variant carriers and their associated gender, diagnosis, age at diagnosis, variant type and most common co-occurring somatic variants including percentages. AML, acute myeloid leukemia; CMML, chronic myelomonocytic leukemia; FPD, familial platelet disorder not otherwise specified; MDS, myelodysplastic syndrome; Runt, RUNX1 homology domain; RunxI, RUNX1 inhibition domain (aka transactivation domain). This schematic was created using cBioPortal (https://www.cbioportal.org/) (68, 69).

Based on literature research including all published patients with a causative *RUNX1* germline variant (15, 34, 115, 132–170), most germline variants are LOF variants, including nonsense, frameshift, and canonical splice variants (n=244, 53.6%) and roughly one third of these variants are located in the C-terminal transactivation domain predicted to not undergo NMD but instead promoting decreased transactivation capacity (**Figure 3A**). Strikingly, CNVs account for 15.4% (n=70) of patients, including whole-gene deletions and both in-frame and out-of-frame recurrent intragenic deletions and duplications. A recurrent deletion of exons 1 and 2 or exons 1 to 3 (NM_001754.5), removing the N-terminal 20 to 33 amino acids of isoform C together with its distal promotor, has been detected in multiple unrelated families with a classic phenotype (37, 149, 155, 168, 171–174). Intragenic duplications are rare but have been independently discovered in two families (135, 169). Nonsynonymous substitutions comprise 29.9% (n=132) of all variants and are mostly located within the RHD that is essential for DNA binding (**Figure 3A**). Causative nonsynonymous variants are primarily found within amino acids 89 to 204 of the RHD, which is where the β-sheet portion of the core binding factor β heterodimerization domain starts, noted as functionally important. Hotspots within the RHD consist of amino acids R107, K110, A134, R162, R166, S167, R169, G170, K194, T196, D198, R201, and R204 (**Figure 3A**) (129, 175). Outside of these amino acid hotspots, most other variants are private and only occur in one index patient/family (**Figure 3B**). Germline indels and noncanonical splice variants are infrequent. A germline translocation t(16;21)(p13;q22) has been described, translocating the distal promotor used for isoform C and the +23 enhancer to chromosome 16 (176). The breakpoint is localized in intron 1 of *RUNX1* with a high content of simple tandem repeats, consistent with the major breakpoint pattern in the recurrent somatic t(12;21) in ALL (177). Another germline translocation t(11;21)(q13;q22) is also primarily affecting isoform C with the breakpoint located within the same chromosomal region of 21q22 (178).

Most germline *RUNX1*-related malignancies are myeloid malignancies, in particular MDS, CMML, and AML that account for 37.3% (n=170) of patients (**Figure 3B**). Given its rarity in a sporadic population, T-ALL (4.4%, n=20) is overrepresented in these patients and has also been established as part of the phenotype (129, 179). Few cases diagnosed with B-ALL (137, 163, 167), non-Hodgkin lymphoma (37, 180), MPN (35), chronic lymphatic leukemia (181) or eosinophilic leukemia (155) have been identified but a causative relationship has not been established (**Figure 3B**). The median age at diagnosis is 42 years for MDS/AML and 22 years for T-ALL (**Figure 3B**).

Concomitant somatic variants frequently occur on the other *RUNX1* allele (26% of patients) as bi-allelic inactivation and as discussed earlier, increase the pre-test probability of a germline allele (**Figure 3B**, **Table 2**). Other frequent co-occurring somatic variants were detected in *TET2* (15%), *FLT3* as ITD or tyrosine kinase domain variant (12%), *SRSF2* (12%), *ASXL1* (11%), *WT1* (8%), *DNMT3A* (8%), *BCOR* (8%) and *BCORL1* (8%) (**Figure 3B**, **Table 2**). Early-onset CHIP has been previously described in *RUNX1* variant carriers and preleukemic individuals were found to carry variants in *DNMT3A*, *TET2, KMT2A, KRAS* and *U2AF1*, consistent with CHIP (37, 139, 153, 174, 182, 183). Recurrent *CDC25C* variants were reported in ~50% of *RUNX1*-mutated patients and hierarchical architecture analysis showed that these variants represent an early event during transformation, defining a pre-leukemic clone (140). Other studies have frequently detected variants in *TET2, BCOR*, *PHF6, CDC25C, SRSF2*, and *GATA2* (13, 37, 184). In one study, *BCOR* variants were particularly common with up to four different variants per patients, however, presence, number or VAF did not correlate with clonal evolution or disease progression (185). Upon cytogenetic analyses, a normal karyotype was most frequently detected, followed by monosomy 7/del(7q), translocations with no apparent recurrent breakpoints/translocation partners and trisomy 8 (**Table 2**). Only few patients were investigated cytogenetically prior to the development of a hematologic malignancy.

## Conclusion

Germline variants predisposing to inherited hematologic malignancies are detected more and more frequently due to increased recognition and detection using NGS-based diagnostic/prognostic panels. A multi-tier approach of several different criteria can be utilized to identify patients with a probable germline variant: (1) germline VAF, (2) bi-allelic inactivation, (3) longitudinal persistence of a pathogenic/likely pathogenic variant at germline VAF when remission is achieved, (4) variants that only occur in germline, and — in the case of *MBD4* variants — (5) significantly higher mutational burden. The clone size in relationship to the blast count, the frequency of somatic variants in the gene in question, and whether the variant has been previously reported as somatic variant and/or is consistent with the mechanism of disease may also be considered (66).

Germline variants in *DDX41*, *ETV6*, *GATA2*, and *RUNX1* have been well-described and may be uncovered upon initial diagnostic/prognostic work-up and subsequently confirmed in appropriate germline material. Importantly, the spectrum of germline predisposition alleles has been expanded over the past few years with the advent of whole-exome or whole-genome sequencing or NGS panel-based sequencing covering non-coding areas and functional testing of variants of unknown significance (VUS). CNVs are frequently identified as causative alleles in patients with RUNX1- and GATA2-deficiency at a frequency of 15.4% and 3.2% respectively, and few families with intragenic deletions of *DDX41* and *ETV6* have been reported as well (67, 84, 89). Germline translocations are infrequent but occur in genes that are otherwise known as common translocation partners in the leukemic clone, namely *ETV6* (86) and *RUNX1* (176, 178) and disrupt the promotor region. Other variants such as noncanonical splice variants and the recurrent variants in the deep-intronic enhancer element of *GATA2* may be not detected or easily overlooked and require functional testing to establish pathogenicity. When suspicion is high based on family history and/or phenotypic criteria, upfront negative testing should be critically questioned, and further testing or re-analysis of the sequencing data considered.

Specific molecular or cytogenetic drivers have been identified and associated with distinct germline syndromes: The development of myeloid disease in *DDX41* germline mutated patients most often requires bi-allelic inactivation of the *DDX41 trans* allele through nonsynonymous substitutions primarily within the helicase domain. Patients are more prone to have a normal karyotype and other recurrent, WHO-subgroup defining aberrations are exceedingly rare. Germline variants in *ETV6*, most often associated with B-ALL, have a normal or hyperdiploid karyotype that is consistent with this type of malignancy. Specific drivers of disease are not apparent but the number of patients with reported molecular/cytogenetic data is small. GATA2-deficiency displays a significant correlation with *ASXL1*-, *SETBP1*- and *STAG2* variants, monosomy 7/del(7q), trisomy 8, and der(1;7)(q10;p10). The der(1;7)(q10;p10) is a specific aberration that only occurs in 0.4% of children and adolescents with MDS and wild-type *GATA2* (109) and may be used as an indicator of a germline *GATA2* variant. Lastly, a second somatic hit of the other allele is also common in patients with germline *RUNX1* variants and somatic variants in *BCOR*, *BCORL1* and *CDC25C* may be more prevalent in these patients. CHIP affecting genes such as *DNMT3A*, *TET2, KMT2A, KRAS* and *U2AF1* and recurrent variants in *CDC25C* may represent early markers of clonal evolution and disease progression in carriers of a *RUNX1* germline variant. Generally, early- versus late somatic alterations in the process of leukemic transformation are not well-studied and patients without obvious hematologic malignancy often do not receive a baseline bone marrow biopsy. Longitudinal pre-leukemic data are needed to identify early markers of clonal evolution and disease progression.

An emergent discussion is whether co-occurring somatic alterations can be used to predict the presence of a germline syndrome and help determining the pathogenicity of the variant when it is formally classified as a VUS. The above-mentioned markers may be applied in a multi-tier approach in addition to the VAF, bi-allelic inactivation, longitudinal persistence of variants and other factors to assess the likelihood of a germline variant in *DDX41*, *ETV6*, *GATA2* and *RUNX1*. Most somatic alterations are not specific enough to be employed within the framework of American College of Medical Genetics and Genomics/Association for Molecular Pathology rules for germline variant interpretation (39). As an exception, bi-allelic *DDX41* inactivations with nonsynonymous substitutions affecting hotspot amino acids or the presence of a der(1;7)(q10;p10) in patients with suspected GATA2-deficiency might confer enough significance, however, this would possibly only apply in combination with other phenotypic features.

In summary, this review provides new insights into the identification of germline syndromes by means of diagnostic/prognostic NGS data as well as specificity and pattern of somatic drivers in patients with germline *DDX41*, *ETV6*, *GATA2*, and *RUNX1* variants based on large patient cohorts. These data will help to predict the clinical course of disease and thereby improve and individualize the clinical management for these patients.

## Author contributions

SF designed and coordinated the study. JZ and DT provided conceptional input. JZ, DT and SF collected, analyzed and interpreted the data. JZ visualized the data. SF wrote the manuscript. All authors contributed to the article and approved the submitted version.
