# Supplementary figures and images for: Germline and somatic drivers in inherited hematologic malignancies
Source: Front Oncol. 2023 Oct 13;13:1205855. doi: 10.3389/fonc.2023.1205855 (PMC10613526; doi:10.3389/fonc.2023.1205855)

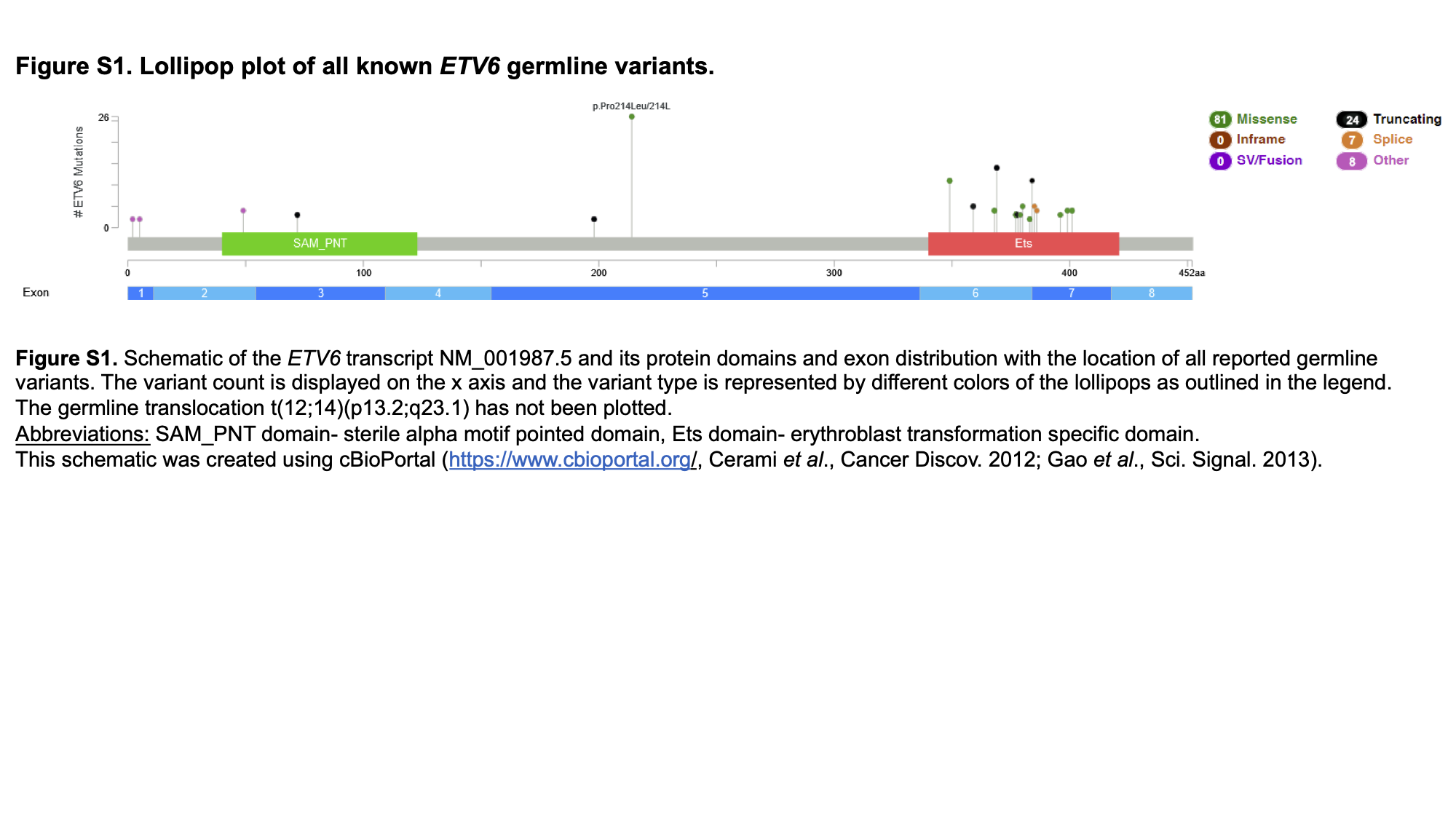

Supplement: Supplementary file 1 [file Image_1.tiff]
